# Alzheimer’s Disease and Different Types of Cancer Likelihood: Unveiling Disparities and Potential Protective Effects in a Korean Cohort Study

**DOI:** 10.3390/cancers15184615

**Published:** 2023-09-18

**Authors:** Ho Suk Kang, Ji Hee Kim, Hyun Lim, Joo-Hee Kim, Hye-Mi Noh, Hyo Geun Choi, Kyueng-Whan Min, Nan Young Kim, Mi Jung Kwon

**Affiliations:** 1 Division of Gastroenterology, Department of Internal Medicine, Hallym University Sacred Heart Hospital, Hallym University College of Medicine, Anyang 14068, Republic of Korea; hskang76@hallym.or.kr (H.S.K.); hlim77@hallym.or.kr (H.L.); 2 Department of Neurosurgery, Hallym University Sacred Heart Hospital, Hallym University College of Medicine, Anyang 14068, Republic of Korea; kimjihee.ns@gmail.com; 3 Division of Pulmonary, Allergy, and Critical Care Medicine, Department of Medicine, Hallym University Sacred Heart Hospital, Hallym University College of Medicine, Anyang 14068, Republic of Korea; luxjhee@gmail.com; 4 Department of Family Medicine, Hallym University Sacred Heart Hospital, Hallym University College of Medicine, Anyang 14068, Republic of Korea; hyeminoh@hallym.or.kr; 5 Suseo Seoul E.N.T. Clinic and MD Analytics, 10, Bamgogae-ro 1-gil, Gangnam-gu, Seoul 06349, Republic of Korea; mdanalytics@naver.com; 6 Department of Pathology, Uijeongbu Eulji Medical Center, Eulji University School of Medicine, 712, Dongil-ro, Uijeongbu-si 11759, Republic of Korea; kyueng@gmail.com; 7 Hallym Institute of Translational Genomics and Bioinformatics, Hallym University Medical Center, Anyang 14068, Republic of Korea; honeyny78@gmail.com; 8 Division of Neuropathology, Department of Pathology, Hallym University Sacred Heart Hospital, Hallym University College of Medicine, Anyang 14068, Republic of Korea; 9 Laboratory of Brain and Cognitive Sciences for Convergence Medicine, Hallym University College of Medicine, Anyang 14068, Republic of Korea

**Keywords:** Alzheimer’s disease, cancer, longitudinal follow-up study, national health screening cohort

## Abstract

**Simple Summary:**

This study investigates the potential link between Alzheimer’s disease (AD) and the probability of developing various cancers using a large cohort dataset in Korea from matching 24,664 Korean citizens with AD and without a history of cancer with 98,656 non-AD and noncancer Korean citizens. The results revealed that patients with AD had a significantly lower likelihood of developing overall malignancy as well as specific types of cancer compared to the control group. Notably, pancreatic cancer showed the strongest inverse relationship with AD. The protective effect against certain organ-specific cancers persisted throughout the 16-year follow-up period, particularly in individuals aged 60 years and above. These findings suggest that Korean patients with AD may have a decreased risk of malignancy, highlighting the need to explore the connection between AD and cancer risk.

**Abstract:**

The link between Alzheimer’s disease and cancer risk is a concern in public health. However, research has yielded limited and sometimes contrasting results, suggesting the need for more validation. We analyzed a large cohort to examine the long-term association between Alzheimer’s disease (AD) and the risk of developing cancer. In total, 24,664 AD patients and 98,656 control participants were selected from the National Health Insurance Cohort database of Korea, spanning from 2002 to 2019. Propensity score matching and overlap-weighted adjustment techniques were used to balance the standardized differences between the AD and control groups. The Cox proportional hazards model was applied to calculate hazard ratios (HRs) with 95% confidence intervals (CIs) for various cancers, considering relevant covariates. Results indicated that patients with AD had a significantly lower likelihood of overall malignancy (HR 0.63; 95% CI, 0.59–0.68) and each of the 10 site-specific cancers compared to the control group. Among these, pancreatic cancer (HR, 0.50) exhibited the strongest inverse association, followed by hepatic (HR, 0.60), gastric (HR, 0.63), kidney (HR, 0.63), lung (HR, 0.64), thyroid (HR, 0.65), colorectal (HR, 0.67), gallbladder and biliary duct (HR, 0.73), hematologic malignancy (HR, 0.73), and bladder cancers (HR, 0.76). This protective effect against certain organ-specific cancers persisted over the 16-year follow-up period, except for in kidney cancer and hematologic malignancies. The protective effect against specific cancer types (gastric, colorectal, lung, hepatic, and pancreatic) was more prominent in individuals aged 60 years and older, regardless of their sex. However, there were some variations in the specific types of cancer observed between males and females. In summary, Korean patients with AD had a lower risk of cancer, especially in individuals 60 years and older, during the 16-year follow-up period.

## 1. Introduction

Alzheimer’s disease (AD) and cancer are two significant health concerns affecting the elderly, particularly in countries with aging populations like Korea [1]. As the geriatric population continues to expand, there has been a concurrent escalation in the occurrences of both cancer and AD [1]. The prevalence of AD exceeds 13% among individuals over 65, with a range of 35% to 50% in the demographic above 85 years [2]. By 2050, it is projected that the combined total of individuals impacted by AD and related dementias will rise to 152 million [3], while a similar timeline anticipates a twofold increase in new cancer cases among older adults to 14 million by 2035, constituting almost 60% of the worldwide cancer incidence [4]. Despite their apparent differences, the relationship between AD and cancer has drawn attention due to emerging research indicating a possible inverse connection between these two disorders [5,6]. AD is linked to extensive neuritic and synaptic degeneration and neuronal cell death, either induced by or concurrently with the deposition of abnormal β-amyloid and tau [7,8]. In contrast, cancer is characterized by disruptions in cellular regulatory mechanisms that enhance cell survival and/or proliferation [9]. One plausible explanation for this inverse relationship is the potential shared malfunction of an underlying mechanism that controls both cell survival and death, contributing to the development of both conditions [9].

Due to the frequent exclusion of elderly individuals from randomized controlled trials, evidence is insufficient concerning the association between AD and cancer risk within this demographic [10]. While previous studies have predominantly focused on the link between neurodegenerative diseases and cancer, with Parkinson’s disease often taking the spotlight as the second most prevalent neurodegenerative disorder after AD, the specific relationship between AD and cancer remains less explored [5,6,11,12]. Notably, investigations into the connection between AD and cancer are limited, with fewer than 10 primary studies available for reference [13,14,15,16,17,18,19,20]. One particularly noteworthy study conducted in the USA uncovered a significant finding: older white adults with AD exhibited a reduced risk of subsequent cancers by 69% (95% confidence interval (CI), 0.12–0.86) [13]. Interestingly, this association was not mirrored in cases of vascular dementia, implying that the relationship between AD and cancer extends beyond mere cognitive impairment or selection bias, suggesting the involvement of shared mechanisms related to altered cellular regulation [13]. Another investigation, utilizing data from the Framingham Heart Study, reported a 61% decrease in the risk of incident cancer among patients with AD (95% CI, 0.26–0.58) [15]. However, due to the limited number of cancer cases available for analysis, this study did not delve into specific cancer types or stratify its findings by age or sex. Despite these gaps in the research, the accumulating evidence underscores the intriguing interplay between AD and cancer, hinting at the potential interwoven mechanisms that underlie these seemingly distinct conditions.

While subsequent studies have provided additional support for the relationship between AD and reduced cancer risk, several limitations have impeded the clarity of results [16,20]. These studies, often characterized by uneven sample sizes [17,18,19], primarily centered on community-based cohorts. This focus on specific cohorts could potentially introduce selection biases stemming from urban or rural disparities, as well as variations in socioeconomic and educational backgrounds [16,20]. These studies did not adequately control for potential confounding variables and biases inherent in their study designs [16,19,20]. One previous population-based study conducted in Korea extensively investigated the incidence of various types of cancer in patients with AD, stratifying the data by age and sex [19]. However, interpreting the results might be limited due to uneven baseline characteristics between the study and control cohorts [19]. Such heterogeneity in baseline characteristics across cohorts can distort the generalizability of findings [21]. Meta-analyses, encompassing 7 and 22 respective studies, observed a significant variance in outcomes, with AD patients displaying a broad range of 6%–40% lower likelihood of developing cancer [22,23]. This variance stemmed from the substantial heterogeneity among these individual studies [22,23]. Given the potential shared risk factors or reciprocal associations between AD and cancer [24], it becomes evident that further validation studies characterized by precisely matched and well-balanced cohort designs are imperative. These designs would serve to adjust for potential mutual confounding factors adequately.

Conducting extensive, detailed, and nationally representative studies that consider the impact of other factors that could influence the results could yield significant evidence regarding the link between AD and cancer occurrence. The primary assumption of this study was that AD might decrease the risk of cancer in general. However, the outcome could vary depending on the specific type of cancer and the age and sex of the individual. To investigate this further, we expanded our study beyond previous research by comparing the well-balanced cohort data with those of the 16-year follow-up using recent nationwide healthcare data that may reflect more recent changes in cancer trends. These variables were adjusted to estimate the potential relationship between AD and the incidence of various site-specific or overall cancers to minimize the confounding effects of demographic data, lifestyle factors, and chronic diseases.

## 2. Patients and Methods

This study used data from the Korean National Health Insurance Service—Health Screening Cohort (KNHIS-HSC) database, a comprehensive source of information for policy and academic investigations. Commencing in 1999, the Korean National Health Insurance Service (KNHIS) has extended compulsory health insurance coverage to approximately 97% of the nation’s populace, with the remaining 3% benefiting from medical aid programs. Pertinently, the KNHIS-HSC sample cohort was originally constituted from individuals who engaged in health screenings during the years 2002 and 2003. This initial participant group, falling within the age bracket of 40 to 79 in 2002, underwent tracking until 2019 [25]. Notably, this cohort comprised 514,866 individuals, meticulously handpicked via a 10% simple random sampling approach from the entire health screening participants during the aforementioned years [25]. Information in the KNHIS-HSC database was anonymized by scrambling the identification codes. The diagnostic codes followed the International Classification of Diseases, 10th Revision, Clinical Modification (ICD-10-CM). A more detailed explanation of KNHIS-HSC data can be found in previous descriptions [25,26,27]. 

This study was conducted in accordance with the guidelines and regulations of the Ethics Committee of Hallym University, and it received approval from the committee (reference number: 2019-10-023). The Institutional Review Board granted a waiver for written informed consent.

### 2.1. Definition of Alzheimer’s Disease

In this study, individuals were classified as having AD if they received a diagnosis of Alzheimer’s (G30) or dementia in Alzheimer’s (F00), but only if they were visited on two or more occasions for the same diagnosis to ensure diagnostic accuracy [27].

For each patient with AD, the index date was defined as the exact day the diagnostic ICD-10 codes for AD were electronically assigned to them in the health insurance claim database. In the case of the non-AD control group, their index date corresponded to the same date as their matched AD patient’s index date.

### 2.2. Definition of Cancers

In this study, the incidence of the following 10 types of cancer was investigated: gastric cancer (ICD-10 codes C16.0–C16.9), malignant neoplasm of cardia [C16.0], fundus of the stomach [C16.1], the body of stomach [C16.2], pyloric antrum [C16.3], pylorus [C16.4], lesser curvature of the stomach, unspecified [C16.5], greater curvature of the stomach, unspecified [C16.6], overlapping sites of the stomach [C16.8], malignant neoplasm of the stomach, unspecified [C16.9]), thyroid cancer (ICD-10 codes: C73), colorectal cancer (ICD-10 codes: C18 to C21 and D010 to D013), lung cancer (ICD-10 codes: C34 and D022), hepatic cancer (ICD-10 codes: C22 and D015), bladder cancer (ICD-10 codes: C67 and D090), pancreatic cancer (ICD-10 codes: C25 and D017), gallbladder and biliary duct (ICD-10 codes: C23 and C24), kidney cancer (ICD-10 codes: C64), and hematologic malignancy (ICD-10 codes: C81 to C96). Individuals assigned the same ICD-10 codes for their specific cancer type on more than two occasions and with more than two clinic visits were considered to have cancer [28,29,30]. The occurrence dates of incident cancers during the follow-up period in both the AD and control groups encompass the timeframe from each participant’s index date until 31 December 2019.

### 2.3. Participant Selection

This study selected 37,427 individuals with AD from a KNHIS-HSC dataset of 514,866 participants, with 895,300,177 medical claim codes recorded from 2002 to 2019. Those already diagnosed with AD in 2002 were excluded after a 1-year washout period (*n* = 174) to ensure that only people newly diagnosed with AD were included. Additionally, individuals with missing records for either BMI or total cholesterol (*n* = 23) and those diagnosed with cancer before their AD diagnosis (*n* = 3306) were excluded from the analysis. The control cohort comprised individuals not diagnosed with AD between 2002 and 2019 (*n* = 477,439). Individuals diagnosed with G30 or F00 according to the ICD-10 codes were excluded (*n* = 7728) to ensure that the control group was free of AD.

**Figure 1:**
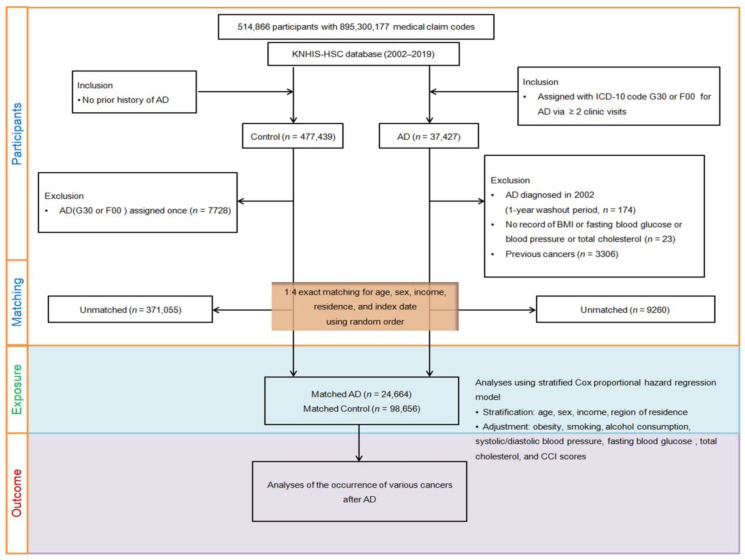
A schematic illustration of the participant selection process used in this study. Overall, 24,664 patients with Alzheimer’s disease (AD) were matched with 98,656 control participants for age, sex, income, and region of residence of the 514,866 participants in the Korean National Health Insurance Service—Health Screening Cohort (KNHIS-HSC) database.

Furthermore, individuals with AD were matched with control participants in a 1:4 ratio based on age, sex, income, and region of residence to reduce the effect of potential confounding factors. To avoid selection bias, control participants were randomly selected and then chosen from top to bottom to match the evaluation date of each participant with AD. It was assumed that the matched control participants were being evaluated simultaneously as each matched AD participant (index date). Therefore, the participants in the control group who died before the index date were discounted. Individuals in both the AD and counterpart groups with a history of cancer before the evaluation date were excluded. During the matching procedure, 9260 participants and 371,055 control participants were excluded. Finally, 24,664 participants with AD were matched with 98,656 counterpart participants in a 1:4 ratio (Figure 1).

Subsequently, the study investigated the incidence of various cancers identified by the corresponding ICD-10 codes in both the AD and control groups from the index date until the end of 2019.

### 2.4. Covariates

The data obtained from the closest date to the index date were considered for analysis. The participants were grouped into 10 different age categories, each with a 5-year interval ranging from 40 to 44 years to 85 years or older. The income levels of the participants were classified into five categories, with Classes 1 and 5 being the lowest and highest incomes, respectively. Residential locations were categorized into 16 groups based on administrative districts and subsequently reorganized into urban or rural areas [31].

The study categorized tobacco smoking based on the current smoking status of the participants, who were classified as nonsmokers, past smokers, or current smokers [27]. Alcohol consumption was categorized based on frequency into two groups: less than once a week and once or more weekly. Obesity was measured using BMI in kg/m^2^ and categorized according to the Asia-Pacific criteria following the Western Pacific Regional Office 2000 [32]. The BMI categories were as follows: underweight (<18.5), normal (≥18.5 to <23), overweight (≥23 to <25), obese I (≥25 to <30), and obese II (≥30). Systolic blood pressure (SBP) and diastolic blood pressure (DBP) were measured in millimeters of mercury (mmHg), fasting blood glucose in milligrams per deciliter (mg/dL), and total cholesterol in milligrams per dL.

The Charlson Comorbidity Index (CCI) was utilized to assess the disease burden by evaluating the severity and number of 17 comorbidities. A score was assigned to each participant based on the presence and severity of the comorbidities, and the CCI was measured as a continuous variable ranging from 0 (no comorbidities) to 29 (multiple comorbidities) [33,34]. This study excluded cancers and metastatic cancers from the CCI score calculations.

### 2.5. Statistical Analyses

In this study, propensity score overlap weighting was employed to ensure a balance between covariates and maximize the effective sample size. A method called propensity score was utilized in this study, which involved a multivariable logistic regression model that considered all relevant factors. Furthermore, to determine the overlap weighting in this study, individuals with AD were assigned weights based on the likelihood of their propensity score, whereas the control group’s weights were based on the probability of one minus their propensity score. Overlap weighting was used since it achieves an exact balance and maximizes precision, with values ranging from 0 to 1. To assess the variation in general characteristics between the AD and control groups, researchers compared the standardized differences in covariates before and after weighting. The matching accuracy was assessed by comparing the absolute standardized differences of covariates before and after matching, with values < 0.20 considered an appropriate balance [35]. The percentages of categorical data were condensed, and the standardized differences were employed to compare the prevalence of general characteristics between the groups in the cohort.

In this study, the cumulative probability of incident cancers in the AD group was compared with that in the control group using Kaplan–Meier analysis and the log-rank test during the follow-up period. An overlap-weighted Cox proportional hazard regression model was used to calculate the hazard ratios (HRs) with 95% CIs for all and various cancers. The incidence and incidence rate differences were also calculated for all outcomes using the crude model and two adjusted models. The analyses were stratified by matching the variables age, sex, income, and region of residence. Subgroup analyses were conducted using a stratified Cox proportional hazards model to compare participants younger than 60 years with those 60 years or older and to compare males and females. Two-tailed analyses were performed, and statistical significance was set at *p* < 0.05. All statistical analyses were performed using SAS version 9.4 (SAS Institute Inc., Cary, NC, USA).

## 3. Results

Table 1 shows the general characteristics of the study participants before and after the overlap weighting adjustment. This study included 24,664 individuals with AD and 98,690 individuals without AD, matched for age, sex, income, and region of residence between 2003 and 2019. The standardized mean differences in age, sex, income, and region of residence between the groups were the same (standardized difference = 0.00). Before implementing the overlap weighting modification, there were slight imbalances in the initial characteristics of the two cohorts. These imbalances exist in obesity status, dyslipidemia, hemoglobin and fasting blood glucose levels, blood pressure, smoking and alcohol habits, CCI score, and total cholesterol levels. Following the implementation of the overlap weighting adjustment, the standardized differences in all covariates were minimized to less than 0.2, indicating a balanced distribution of characteristics between the AD and control groups.

### 3.1. Association of Occurrence of Malignancy between the Group with AD and the Controls

Table 2 shows the crude and adjusted HRs for AD in various cancers. The incidence rates of cancer for the entire study population were 11.63 and 16.48 per 1000 person-years for the AD and control groups, respectively. The top five types of cancer observed in the group with AD were lung cancer (2.57 incidence rate per 1000 person-years), followed by gastric cancer (2.33), colorectal cancer (2.27), hepatic cancer (1.41), and gallbladder and biliary duct cancer (0.87). Compared with controls, the patients with AD showed incidence rate differences in the events of gastric cancer (−1.02), thyroid cancer (−0.22), colorectal cancer (−0.98), lung cancer (−1.19), hepatic cancer (−0.32), bladder cancer (−0.22), pancreatic cancer (−0.50), gallbladder and biliary duct cancer (−0.26), kidney cancer (−0.11), and hematologic malignancy (−0.17), which indicated a lower trend in the development of various site-specific cancers in the patients with AD.

In the unadjusted model, the HRs for overall cancer risk, as well as specific cancer types including gastric, thyroid, colorectal, lung, hepatic, bladder, pancreatic, gallbladder and biliary duct, kidney, and hematologic malignancy, were 0.70, 0.68, 0.63, 0.69, 0.69, 0.81, 0.79, 0.57, 0.78, 0.72, and 0.81, respectively.

Upon controlling for confounding factors such as age, sex, income, region of residence, obesity, smoking, alcohol consumption, CCI scores, SBP, DBP, fasting blood glucose, and total cholesterol, the HRs for the overall incidence of cancer, as well as specific cancer types, including gastric, thyroid, colorectal, lung, hepatic, bladder, pancreatic, gallbladder and biliary duct, kidney, and hematological malignancy, remained statistically significant with HRs of 0.63, 0.63, 0.65, 0.67, 0.64, 0.60, 0.76, 0.50, 0.73, 0.63, and 0.73, respectively. This result indicated a statistically significant association between cancer (including overall malignancy and all 10 site-specific cancers) and a reduced risk of AD, compared to the control group.

**Figure 2:**
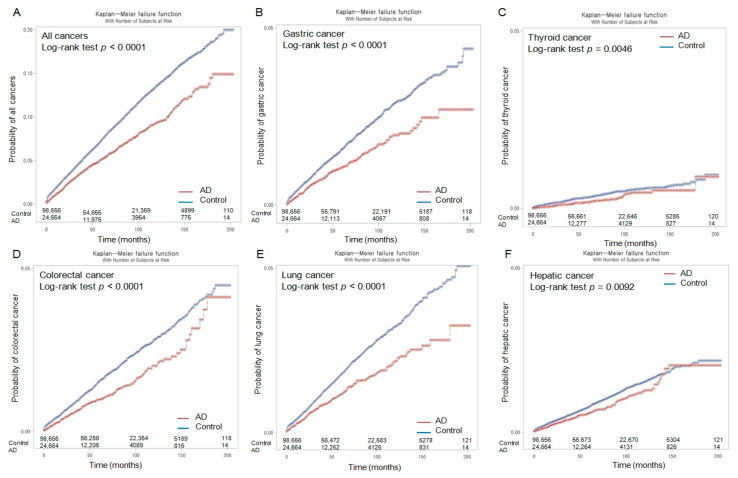
Kaplan–Meier incidence probability of overall malignancy (**A**), gastric cancer (**B**), thyroid cancer (**C**), colorectal cancer (**D**), lung cancer (**E**), and hepatic cancer (**F**) in patients with Alzheimer’s disease (AD) and control populations over 16 years from the index date.

**Figure 3:**
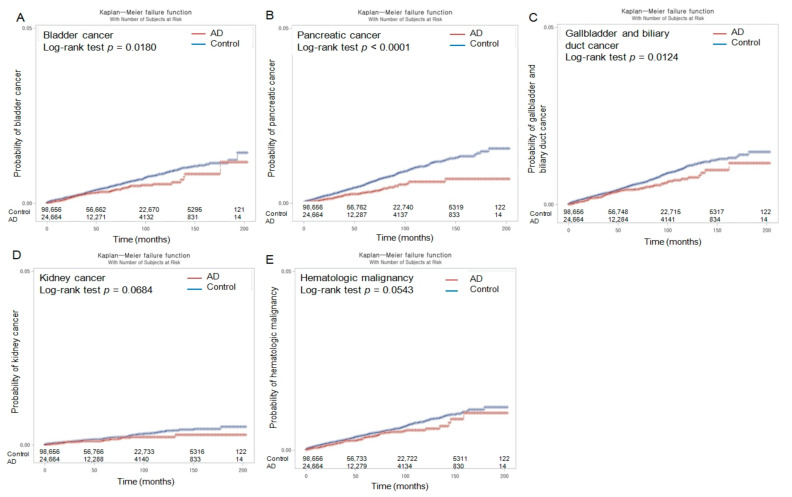
Kaplan–Meier incidence probability of bladder cancer (**A**), pancreatic cancer (**B**), gallbladder and biliary duct cancer (**C**), kidney cancer (**D**), and hematologic malignancy (**E**) in patients with Alzheimer’s disease (AD) and control populations over 16 years from the index date.

Throughout the 16-year follow-up period, the Kaplan–Meier analyses and log-rank tests revealed lower probabilities of developing overall malignancies (*p <* 0.0001) and eight site-specific cancer types (gastric (*p <* 0.0001), thyroid (*p =* 0.0046), colorectal (*p <* 0.0001), lung (*p <* 0.0001), hepatic (*p =* 0.0092), bladder (*p =* 0.0180), pancreatic (*p <* 0.0001), and gallbladder and biliary duct (*p =* 0.0124) cancers) in the patients with AD than in those in the control group (Figure 2 and Figure 3). 

However, the incidence probabilities of developing kidney cancer and hematologic malignancy did not exhibit a significant difference between the AD and control groups (with *p*-values of 0.0684 and 0.0543, respectively) throughout the follow-up period.

### 3.2. Subgroup Analysis According to Sex

**Figure 4:**
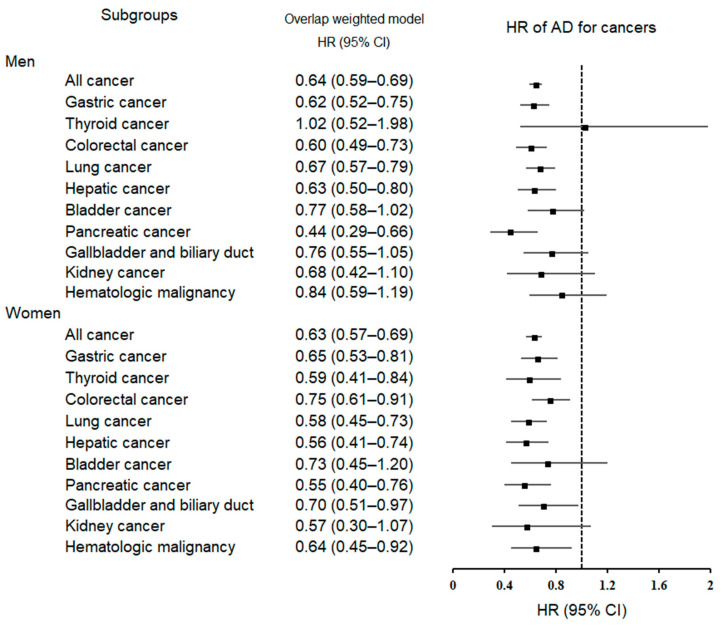
A forest plot depicting the association between Alzheimer’s disease (AD) and subsequent risk of various cancers in men and women. Abbreviations: HR, hazard ratio; 95% CI, 95% confidence interval.

To examine the connection between AD and the emergence of specific types of cancer, we categorized the patients based on their age and sex since these factors are correlated with the incidence of both AD and cancer. In the subgroup analyses according to sex (Table 3; Figure 4) for men, the HRs for overall malignancy, gastric cancer, colorectal cancer, lung cancer, hepatic cancer, and pancreatic cancer were statistically significant after full adjustment (all *p <* 0.05).

In women, the HRs for overall malignancy, gastric cancer, thyroid cancer, colorectal cancer, lung cancer, hepatic cancer, pancreatic cancer, gallbladder and biliary duct cancer, and hematologic malignancy were statistically significant after full adjustment (all *p <* 0.05).

### 3.3. Subgroup Analysis According to Age

In the subgroup analyses according to age (Table 4), no significant association was found between cancer and the risk of AD in individuals aged <60 years. 

For individuals aged ≥60 years, the HRs for overall malignancy and all 10 types of organ-specific cancers (gastric, thyroid, colorectal, lung, hepatic, bladder, pancreatic, gallbladder, biliary duct, kidney cancer, and hematologic malignancy) were statistically significant in all models (all *p* < 0.05). 

## 4. Discussion

Our nationwide cohort study indicated a statistically meaningful diminution in the likelihood of overall malignancy and all 10 site-specific cancers evaluated (gastric, thyroid, colorectal, lung, hepatic, bladder, pancreatic, gallbladder, biliary duct, kidney, and hematologic malignancies) in the AD group compared to the counterpart group. This trend persisted throughout the 16-year follow-up period, indicating that the reduced likelihood of subsequent cancer development in the AD group was sustained for overall malignancy and eight organ-specific cancer types (except for kidney cancer and hematologic malignancies). Further subgroup analyses showed that certain cancers, such as gastric, colorectal, lung, and pancreatic cancers, were less likely to develop in the AD group than in the control group, particularly among males and females aged 60 years or older. 

From a cohort of 24,664 individuals with AD and 98,690 individuals without AD, our analysis revealed that those with AD exhibited a 37% lower probability of developing overall malignancies (HR 0.63; 95% CI, 0.59–0.68) compared to those without AD. This magnitude of effect as indicated by the HR aligns consistently with findings in Western countries, ranging from a minimum 50% risk reduction in an Italian cohort [16] to 61–69% in the primarily white population of the United States [13,15]. Our results also closely align with two large-scale Taiwanese nationwide population-based studies, one involving 6960 patients with AD, indicating a 12% reduction (95% CI, 0.80–0.97) [17], and another with 3282 patients with AD showing a 23% reduced risk (95% CI, 0.65–0.91) [18] of developing overall cancer. Additionally, findings from a prior Korean cohort study encompassing 4408 patients with AD reported a 33% reduction in total cancer hazard (HR 0.67; 95% CI, 0.59–0.75) [19] among those with AD over a 7 to 11-year follow-up period. In parallel, a recent meta-analysis, encompassing 22 studies, bolstered this trend by confirming a statistically significant yet subtle inverse association between AD and cancer. This association appears to withstand scrutiny against potential biases related to confounder handling, diagnostic bias, and competing risks. However, the aspect of survival bias remains inconclusive [23]. Our study supports and extends these findings by demonstrating the correlation between AD and a reduced likelihood of overall malignancy persisting across a 16-year follow-up period. This association remains robust even after considering demographic, socioeconomic, and lifestyle factors and medical comorbidities in our analysis. To address potential biases and enhance the credibility of our findings, we employed a rigorous methodology. Specifically, we utilized propensity scores to match controls from the nationwide population and applied the overlap weighting method for result calibration [36]. Our approach was designed to minimize selection bias and heterogeneity, contributing to the reliability of our results. Utilizing a more extensive nationwide cohort dataset compared to previous studies, our investigation further supports the potential association between AD and a reduced likelihood of developing cancer.

We noted the reduced likelihoods of all 10 listed organ-specific cancers in the patients with AD, with pancreatic cancer (HR, 0.50) displaying the greatest inverse relationship, followed by hepatic (HR, 0.60), gastric (HR, 0.63), kidney (HR, 0.63), lung (HR, 0.64), thyroid (HR, 0.65), colorectal (HR, 0.67), gallbladder and biliary duct (HR, 0.73) cancers, hematologic malignancy (HR, 0.73), and bladder cancer (HR, 0.76). This inverse correlation may not be consistently observed across all types of cancer, as it can vary based on different research studies. Some studies have reported significant risk reductions for certain cancer types, such as colon cancer, lung cancer, melanoma, prostate cancer, and colorectal cancer [16,18,37]. In contrast, others highlight a decreased risk of incident cancer, irrespective of smoking status or smoking-related cancers [15]. For instance, the cancer register of a southern Sweden study reported significant risk reductions for colon cancer, lung cancer, melanoma, and prostate cancer [37]. However, a cohort study conducted in northern Italy found a significant reduction in cancer risk for lung and colorectal cancers [16]. A Taiwanese study also reported significant risk reductions in colon and prostate cancers [18]. However, a cohort study in the USA documented a diminished risk of incident cancer, encompassing both smoking-related and unrelated cancers [15]. 

Furthermore, certain epidemiological studies have even reported a bidirectional inverse relationship. These studies highlighted a lower risk of developing AD in conjunction with specific cancer sites, including colorectal, lung, and breast cancers [15,38,39]. Our research may align with previous investigations in uncovering the reduced risk of colorectal and lung cancers among AD patients. Differences in cancer prevalence and incidence in other countries and cancer type, stage, and treatment methods may account for some of these discrepancies [40]. In our study, lung, gastric, colorectal, hepatic, gallbladder, and biliary duct cancers were the five most common cancers in the AD group, consistent with the most commonly diagnosed cancers in Korea, including thyroid, lung, stomach, and colorectal cancers [41]. The protective effects of AD against incident pancreatic (50% reduction), hepatic (40% reduction), lung (36% reduction), colorectal (33% reduction), and gastric (37% reduction) cancers were clinically significant, particularly given that lung cancer is the leading cause of death attributed to cancer in Korea, followed by hepatic, colorectal, gastric, and pancreatic cancers [41]. Furthermore, Korea has a higher incidence of gastric cancer than other regions worldwide [42]. The negative association between AD and cancer may suggest that the susceptibility to one disease may protect against the other [43], implying a potential reciprocal association between the two diseases [24]. Creating therapies aimed at the shared mechanisms could provide a dual advantage by tackling AD and cancer simultaneously. Several drugs indicated for cancer treatment presented negative correlations with AD profiles, including the aromatase inhibitor exemestane used for the treatment of BRCA, the progestin medication megestrol, the alkylating agent thiotepa, tretinoin, and estradiol [44].

Previous studies have provided limited information regarding the differences in cancer risk associated with age and sex in patients with AD. Our study found a reduced likelihood of overall malignancies and all types of cancers in individuals aged 60 years. This result is in agreement with a study conducted in Taiwan, which suggested that individuals aged 60–79 years were more likely to experience a lower incidence of new cancer diagnoses [17]. When stratified according to sex, the lower probability of overall malignancy in the AD group was consistent across both males and females but with some variation in specific types of cancer. Interestingly, female individuals with AD exhibited a lessened hazard of thyroid, gallbladder, biliary duct, and hematologic malignancies, whereas male patients with AD did not exhibit any site-specific cancers. However, previous studies have reported contradictory findings, possibly due to differences in the inclusion criteria, baseline characteristics, and diagnostic criteria [19,40,45]. For example, in a previous Korean study, head and neck cancer and gastric cancer were reported to be specific to male patients with AD, whereas pancreatic cancer was specific to female patients with AD regarding risk reduction for malignant potential [19]. In a Taiwanese study, male patients with AD were associated only with a lower likelihood of incident lung cancer (odds ratio 0.61; 95% CI, 0.40–0.88) [17]. Moreover, a study from Italy reported a lower risk of general and endocrine-related neoplasms in female patients with AD [14], suggesting a sex-specific and endocrine-related association between benign and malignant tumors. However, this study had limitations, such as the small number of neoplasms and the vague criteria for endocrine-related neoplasms [46].

Although the relationship between these two conditions cannot be completely explained by bias or confounding factors [23,36], both disorders share similar risk factors and coexisting medical conditions, such as increasing age, diabetes, obesity, and metabolic syndrome [38,47], possibly with some common molecular pathways in their pathogenesis [24]. Recent molecular studies suggest that genetic and molecular factors contribute to the association between AD and cancer [44,48,49]. Approximately 70% of AD risk is attributed to genetics [50]. The possible candidate pathways involved with the opposite direction between AD and cancers were found, including MYC targets, mTORC1 signaling, cell cycle checkpoints, DNA repair, unfolded protein response, proteasome, stabilization of p53, myogenesis, KRAS signaling, allograft rejection, and the complement cascade [44]. In one study utilizing the Mendelian randomization analysis approach, a total of 28 genetic variants linked with cancer were identified, and intriguingly, these variants also exhibited a correlation with a reduced likelihood of developing AD [49]. Notably, 13 of these 28 variants are located either directly on or close to genes previously linked to AD [49]. In a more recent investigation, PVRIG’s potential role in suppressing tumors came into focus, with notable positive links between genetic regions associated with AD risk and heightened PVRIG expression [48]. Colocalization analysis further confirmed the impact of increased PVRIG expression on AD risk, a conclusion reinforced by observations revealing its close association with lower stemness scores and favorable correlations with immune responses against tumors and overall survival [48].

## 5. Strengths and Limitations

First, the reliability of this research stemmed from the use of comprehensive, nationally representative data that were adjusted for variables such as socioeconomic status, lifestyle-related factors, and comorbidities that could potentially increase the risk of both AD and cancer. Second, to limit selection bias and increase the study’s accuracy, a t-balanced cohort of 24,664 participants with AD and 98,656 participants without AD was matched using propensity scores, which may mimic randomized trials. Although AD and cancer are both commonly observed in older individuals, the use of a sample of 24,664 individuals with AD that uniformly matched with 98,656 participants without AD within the relevant age categories resulted in a balanced distribution of sex and age in the study. Demographic heterogeneity among participants may have influenced the magnitude of the associations observed between the original characteristics of the research groups [21]. Therefore, using this process, we concluded a potential link between AD and a decreased risk of developing overall malignancy or specific types of cancer in both men and women with AD. Third, as the data were collected from all medical and clinical services in Korea, comprehensive medical histories could be accessed during the follow-up period, increasing the generalizability and credibility of the study results. Additionally, the 16-year follow-up period provided a significant advantage. This is one of the most extensive longitudinal studies of the relationship between AD and malignancies. 

Our study had some limitations. First, confounding factors may not have been considered because this study only included Korean nationals and relied on diagnostic codes. Second, no data existed regarding family history, personal genetics, or diet for AD or cancer in the KNHIS-HSC database; therefore, information gaps were not filled. Third, ascertainment bias should be considered, as individuals with AD are more likely to seek medical care and undergo diagnostic investigations than those without AD. Fourth, it should be noted that the association between cancer and AD is complex and may depend on many factors, including the type and stage of cancer, treatment methods, and other comorbidities. Fifth, assessing HR through age stratification (60–74 and 75+) was not feasible, although it could have been intriguing in differentiating early-onset and late-onset AD. This limitation arose due to the expiration of data accessibility within the NHIS database. The ownership of sample cohort data in NHIS rests outside the authors’ control, requiring researchers to access and analyze the data, as well as export the outcomes, either by visiting the analysis center or by remote means.

## 6. Conclusions

Based on a large representative population adjusted for potential confounding factors, our study demonstrated that patients with AD in the Korean population, particularly those aged ≥60 years regardless of sex, may be less likely to experience either overall malignancy or peculiar cancer types, providing important evidence for the association between AD and incident cancer. The results of our research indicate that AD may offer a level of protection against several forms of cancer, although the underlying mechanisms require further investigation. Given that AD and cancer are substantial physical, emotional, and financial burdens on individuals, their families, and society, an awareness of the potentially lower incidence of cancer among patients with AD may be informative for cancer surveillance and management in this population.

## Figures and Tables

**Table 1:** General characteristics of participants after and before propensity score overlap weighting adjustment.

Characteristics		Before Overlap Weighting Adjustment			After Overlap Weighting Adjustment		
		Dementia (*n* = 24,664)	Control (*n* = 98,656)	Standardized Difference	Dementia (*n* = 24,664)	Control (*n* = 98,656)	Standardized Difference
Age (y), %				0.00			0.00
	40–44	0.01	0.01		0.01	0.01	
	45–49	0.21	0.21		0.20	0.20	
	50–54	0.79	0.79		0.77	0.77	
	55–59	2.57	2.57		2.53	2.53	
	60–64	5.83	5.83		5.75	5.75	
	65–69	13.36	13.36		13.22	13.22	
	70–74	25.25	25.25		25.14	25.14	
	75–79	35.62	35.62		35.78	35.78	
	80–84	15.30	15.30		15.51	15.51	
	85+	1.06	1.06		1.09	1.09	
Sex, %				0.00			0.00
	Male	42.60	42.60		42.11	42.11	
	Female	57.40	57.40		57.89	57.89	
Income, %				0.00			0.00
	1 (lowest)	19.94	19.94		19.62	19.62	
	2	10.72	10.72		10.64	10.64	
	3	13.19	13.19		13.24	13.24	
	4	19.01	19.01		19.04	19.04	
	5 (highest)	37.15	37.15		37.45	37.45	
Region of residence, %				0.00			0.00
	Urban	35.91	35.91		35.82	35.82	
	Rural	64.09	64.09		64.18	64.18	
Obesity, %				0.12			0.00
	Underweight	4.69	3.41		4.38	4.38	
	Normal	38.49	34.46		37.64	37.64	
	Overweight	24.34	26.35		24.89	24.89	
	Obese I	29.22	32.26		29.80	29.80	
	Obese II	3.26	3.51		3.29	3.29	
Smoking status, %				0.06			0.00
	Nonsmoker	77.21	76.97		77.50	77.50	
	Past smoker	11.87	13.46		12.18	12.18	
	Current smoker	10.93	9.57		10.31	10.31	
Alcohol consumption, %				0.07			0.00
	<1 time a week	71.34	68.02		70.72	70.72	
	≥1 time a week	28.66	31.98		29.28	29.28	
Systolic blood pressure (mean, SD)		130.41 (17.56)	130.50 (16.60)	0.01	130.34 (14.77)	130.34 (7.18)	0.00
Diastolic blood pressure (mean, SD)		78.28 (10.94)	78.08 (10.37)	0.02	78.16 (9.20)	78.16 (4.50)	0.00
Fasting blood glucose (mean, SD)		107.85 (37.71)	103.82 (29.07)	0.12	106.22 (29.41)	106.22 (14.41)	0.00
Total cholesterol (mean, SD)		195.41 (42.06)	195.30 (40.26)	0.00	195.40 (35.03)	195.40 (17.77)	0.00
CCI score (mean, SD)		1.84 (1.76)	0.76 (1.19)	0.72	1.43 (1.22)	1.43 (0.71)	0.00
All cancer, %		4.90	7.99	0.13	4.84	8.73	0.15
Gastric cancer, %		0.99	1.66	0.06	0.98	1.76	0.07
Thyroid cancer, %		0.19	0.33	0.03	0.20	0.34	0.03
Colorectal cancer, %		0.97	1.62	0.06	0.99	1.71	0.06
Lung cancer, %		1.11	1.89	0.06	1.12	2.07	0.08
Hepatic cancer, %		0.61	0.87	0.03	0.54	1.09	0.06
Bladder cancer, %		0.32	0.48	0.03	0.32	0.51	0.03
Pancreatic cancer, %		0.29	0.59	0.05	0.30	0.67	0.05
Gallbladder and biliary duct, %		0.38	0.57	0.03	0.38	0.61	0.03
Kidney cancer, %		0.13	0.21	0.02	0.13	0.23	0.02
Hematologic malignancy, %		0.32	0.46	0.02	0.32	0.50	0.03

Abbreviations: CCI, Charlson Comorbidity Index; SD, standard deviation.

**Table 2:** Crude and adjusted hazard ratios of Alzheimer’s disease (AD) for various cancers.

Dependent Variable	IR per 1000 PY		IRD per 1000 PY (95% CI)	Hazard Ratios for Cancers (95% CI)			
	AD (*n* = 3070)	Control (*n* = 12,280)		Crude †	*p*	Model 1 †,‡	*p*
All cancer (*n* = 9087)	11.63	16.48	−4.85 (−5.69 to −4.02)	0.70 (0.66–0.75)	<0.001 *	0.63 (0.59–0.68)	<0.001 *
Gastric cancer (*n* = 1883)	2.33	3.35	−1.02 (−1.40 to −0.65)	0.68 (0.60–0.78)	<0.001 *	0.63 (0.55–0.73)	<0.001 *
Thyroid cancer (*n* = 374)	0.44	0.66	−0.22 (−0.38 to −0.05)	0.63 (0.46–0.85)	0.003 *	0.65 (0.47–0.90)	0.009 *
Colorectal cancer (*n* = 1838)	2.27	3.25	−0.98 (−1.35 to −0.61)	0.69 (0.61–0.80)	<0.001 *	0.67 (0.58–0.77)	<0.001 *
Lung cancer (*n* = 2133)	2.57	3.76	−1.19 (−1.59 to −0.80)	0.69 (0.61–0.79)	<0.001 *	0.64 (0.56–0.73)	<0.001 *
Hepatic cancer (*n* = 1009)	1.41	1.73	−0.32 (−0.59 to −0.05)	0.81 (0.68–0.97)	0.020 *	0.60 (0.50–0.72)	<0.001 *
Bladder cancer (*n* = 557)	0.74	0.96	−0.22 (−0.42 to −0.02)	0.79 (0.62–1.00)	0.051	0.76 (0.59–0.98)	0.031 *
Pancreatic cancer (*n* = 658)	0.68	1.18	−0.50 (−0.72 to −0.29)	0.57 (0.45–0.73)	<0.001 *	0.50 (0.39–0.65)	<0.001 *
Gallbladder and BD (*n* = 654)	0.87	1.13	−0.26 (−0.48 to −0.04)	0.78 (0.63–0.98)	0.029 *	0.73 (0.58–0.92)	0.007 *
Kidney cancer (*n* = 242)	0.31	0.42	−0.11 (−0.24 to 0.02)	0.72 (0.50–1.05)	0.084	0.63 (0.43–0.93)	0.019 *
Hematologic malignancy (*n* = 537)	0.75	0.92	−0.17 (−0.37 to 0.03)	0.81 (0.64–1.02)	0.078	0.73 (0.57–0.94)	0.014 *

Abbreviations: IR, incidence rate; PY, person-years; IRD, incidence rate difference; 95% CI, 95% confidence interval; BD, biliary duct. * Stratified Cox proportional hazards regression model: significance at *p* < 0.05. † Crude model was stratified by age, sex, income, and region of residence. ‡ Model 1 was adjusted for obesity, smoking, alcohol consumption, CCI scores, systolic blood pressure, diastolic blood pressure, fasting blood glucose, and total cholesterol.

**Table 3:** Subgroup analysis of crude and adjusted hazard ratios of Alzheimer’s disease (AD) for various cancers by sex.

Dependent Variable	IR per 1000 PY		IRD per 1000 PY (95% CI)	Hazard Ratios for Cancers (95% CI)			
	AD	Control		Crude †	*p*	Model 1 †,‡	*p*
Men (*n* = AD: 10,506, control: 42,024)							
All cancer (*n* = 5172)	18.43	25.09	−6.66 (−8.40 to −4.92)	0.71 (0.66–0.77)	<0.001 *	0.64 (0.59–0.69)	<0.001 *
Gastric cancer (*n* = 1119)	3.72	5.32	−1.60 (−2.39 to −0.81)	0.67 (0.56–0.80)	<0.001 *	0.62 (0.52–0.75)	<0.001 *
Thyroid cancer (*n* = 69)	0.32	0.3	0.01 (−0.18 to 0.21)	1.04 (0.56–1.94)	0.906	1.02 (0.52–1.98)	0.966
Colorectal cancer (*n* = 981)	2.99	4.66	−1.67 (−2.40 to −0.93)	0.63 (0.51–0.76)	<0.001 *	0.60 (0.49–0.73)	<0.001 *
Lung cancer (*n* = 1439)	4.99	6.66	−1.68 (−2.56 to −0.79)	0.74 (0.63–0.86)	<0.001 *	0.67 (0.57–0.79)	<0.001 *
Hepatic cancer (*n* = 595)	2.45	2.66	−0.21 (−0.78 to 0.35)	0.89 (0.71–1.11)	0.298	0.63 (0.50–0.80)	<0.001 *
Bladder cancer (*n* = 412)	1.55	1.87	−0.32 (−0.79 to 0.15)	0.81 (0.62–1.07)	0.145	0.77 (0.58–1.02)	0.072
Pancreatic cancer (*n* = 293)	0.71	1.41	−0.70 (−1.09 to −0.30)	0.49 (0.33–0.73)	<0.001 *	0.44 (0.29–0.66)	<0.001 *
Gallbladder and BD (*n* = 335)	1.23	1.52	−0.29 (−0.71 to 0.13)	0.81 (0.60–1.10)	0.182	0.76 (0.55–1.05)	0.095
Kidney cancer (*n* = 151)	0.55	0.69	0.11 (−0.71 to 0.93)	0.76 (0.48–1.21)	0.243	0.68 (0.42–1.10)	0.118
Hematologic malignancy (*n* = 266)	1.1	1.19	−0.08 (−0.46 to 0.30)	0.91 (0.65–1.26)	0.556	0.84 (0.59–1.19)	0.316
Women (*n* = AD: 14,158, control: 56,632)							
All cancer (*n* = 3915)	7.9	11.32	−3.43 (−4.29 to −2.56)	0.69 (0.63–0.75)	<0.001 *	0.63 (0.57–0.69)	<0.001 *
Gastric cancer (*n* = 764)	1.56	2.16	−0.60 (−0.98 to −0.22)	0.71 (0.58–0.87)	<0.001 *	0.65 (0.53–0.81)	<0.001 *
Thyroid cancer (*n* = 305)	0.51	0.88	−0.37 (−0.61 to −0.13)	0.55 (0.39–0.78)	0.001 *	0.59 (0.41–0.84)	0.004 *
Colorectal cancer (*n* = 857)	1.87	2.39	−0.52 (−0.92 to −0.13)	0.77 (0.64–0.93)	0.007 *	0.75 (0.61–0.91)	0.003 *
Lung cancer (*n* = 694)	1.23	1.99	−0.76 (−1.12 to −0.40)	0.61 (0.49–0.77)	<0.001 *	0.58 (0.45–0.73)	<0.001 *
Hepatic cancer (*n* = 414)	0.83	1.16	−0.33 (−0.60 to −0.05)	0.71 (0.54–0.95)	0.018 *	0.56 (0.41–0.74)	<0.001 *
Bladder cancer (*n* = 145)	0.29	0.41	−0.11 (−0.28 to 0.05)	0.72 (0.45–1.16)	0.174	0.73 (0.45–1.20)	0.213
Pancreatic cancer (*n* = 365)	0.66	1.04	−0.38 (−0.64 to −0.12)	0.63 (0.46–0.86)	0.003 *	0.55 (0.40–0.76)	0.003 *
Gallbladder and BD (*n* = 319)	0.67	0.89	−0.22 (−0.46 to 0.03)	0.76 (0.55–1.03)	0.080	0.70 (0.51–0.97)	0.033 *
Kidney cancer (*n* = 91)	0.18	0.26	−0.08 (−0.21 to 0.05)	0.67 (0.36–1.23)	0.192	0.57 (0.30–1.07)	0.081
Hematologic malignancy (*n* = 271)	0.56	0.76	−0.20 (−0.42 to 0.02)	0.72 (0.51–1.02)	0.061	0.64 (0.45–0.92)	0.014 *

Abbreviations: IR, incidence rate; PY, person-years; IRD, incidence rate difference; 95% CI, 95% confidence interval; BD, biliary duct. * Stratified Cox proportional hazards regression model: significance at *p* < 0.05. † Crude model was stratified by age, sex, income, and region of residence. ‡ Model 1 was adjusted for obesity, smoking, alcohol consumption, CCI scores, systolic blood pressure, diastolic blood pressure, fasting blood glucose, and total cholesterol.

**Table 4:** Subgroup analysis of crude and adjusted hazard ratios of Alzheimer’s disease (AD) for various cancers by age.

Dependent Variable	IR per 1000 PY		IRD per 1000 PY (95% CI)	Hazard Ratios for Cancers (95% CI)			
	AD	Control		Crude †	*p*	Model 1 †,‡	*p*
Age < 60 (*n* = AD: 250, control: 1000)							
All cancer (*n* = 72)	5.75	6.53	−0.78 (−4.58 to 3.02)	0.88 (0.47–1.64)	0.695	0.68 (0.32–1.48)	0.332
Gastric cancer (*n* = 16)	0.47	1.60	−1.13 (−2.89 to 0.63)	0.31 (0.04–2.34)	0.254	0.20 (0.02–2.16)	0.185
Thyroid cancer (*n* = 9)	0.94	0.74	0.19 (−1.12 to 1.51)	1.25 (0.26–6.01)	0.785	1.33 (0.23–7.72)	0.753
Colorectal cancer (*n* = 12)	0.94	1.06	−0.13 (−1.64 to 1.39)	0.87 (0.19–3.99)	0.859	2.03 (0.28–14.80)	0.486
Lung cancer (*n* = 8)	0.00	0.85	−0.85 (−2.08 to 0.38)	N/A	0.995	N/A	0.994
Hepatic cancer (*n* = 13)	2.36	0.85	1.51 (−0.07 to 3.09)	2.78 (0.91–8.52)	0.074	1.92 (0.30–12.24)	0.490
Bladder cancer (*n* = 3)	0.00	0.32	−0.32 (−1.07 to 0.44)	N/A	0.997	N/A	0.998
Pancreatic cancer (*n* = 3)	0.00	0.32	−0.32 (−1.07 to 0.44)	N/A	0.997	N/A	0.999
Gallbladder and BD (*n* = 1)	0.00	0.11	−0.11 (−0.54 to 0.33)	N/A	0.999	N/A	1.000
Kidney cancer (*n* = 4)	0.00	0.42	−0.42 (−1.30 to 0.45)	N/A	0.997	N/A	1.000
Hematologic malignancy (*n* = 6)	1.40	0.32	1.09 (0.02 to 2.16)	4.27 (0.86–21.17)	0.076	2.87 (0.24–33.99)	0.403
Age ≥ 60 (*n* = AD: 24,414, control: 97,656)							
All cancer (*n* = 9015)	11.75	16.68	−4.93 (−5.78 to −4.08)	0.70 (0.66–0.74)	<0.001 *	0.63 (0.59–0.67)	<0.001 *
Gastric cancer (*n* = 1867)	2.37	3.39	−1.02 (−1.40 to −0.64)	0.69 (0.60–0.79)	<0.001 *	0.64 (0.55–0.73)	<0.001 *
Thyroid cancer (*n* = 365)	0.43	0.66	−0.23 (−0.39 to −0.06)	0.61 (0.45–0.84)	0.002 *	0.64 (0.46–0.88)	0.007 *
Colorectal cancer (*n* = 1826)	2.29	3.29	−1.00 (−1.37 to −0.62)	0.69 (0.61–0.80)	<0.001 *	0.67 (0.58–0.77)	<0.001 *
Lung cancer (*n* = 2125)	2.62	3.82	−1.20 (−1.60 to −0.80)	0.70 (0.61–0.79)	<0.001 *	0.64 (0.56–0.73)	<0.001 *
Hepatic cancer (*n* = 996)	1.39	1.75	−0.36 (−0.63 to −0.08)	0.79 (0.67–0.95)	<0.001 *	0.59 (0.49–0.71)	<0.001 *
Bladder cancer (*n* = 554)	0.76	0.98	−0.22 (−0.42 to −0.01)	0.79 (0.63–1.01)	0.058	0.77 (0.60–0.98)	0.036 *
Pancreatic cancer (*n* = 655)	0.69	1.20	−0.51 (−0.73 to −0.28)	0.57 (0.45–0.73)	<0.001 *	0.51 (0.39–0.65)	<0.001 *
Gallbladder and BD (*n* = 653)	0.89	1.15	−0.26 (−0.48 to −0.04)	0.78 (0.63–0.98)	0.030 *	0.73 (0.58–0.92)	0.008 *
Kidney cancer (*n* = 238)	0.32	0.42	−0.10 (−0.24 to 0.03)	0.74 (0.51–1.07)	0.105	0.65 (0.44–0.96)	0.028 *
Hematologic malignancy (*n* = 531)	0.74	0.93	−0.19 (−0.39 to 0.01)	0.78 (0.62–1.00)	0.048 *	0.71 (0.55–0.91)	0.008 *

Abbreviations: IR, incidence rate; PY, person-years; IRD, incidence rate difference; 95% CI, 95% confidence interval; BD, biliary duct. * Stratified Cox proportional hazards regression model: significance at *p* < 0.05. † Crude model was stratified by age, sex, income, and region of residence. ‡ Model 1 was adjusted for obesity, smoking, alcohol consumption, CCI scores, systolic blood pressure, diastolic blood pressure, fasting blood glucose, and total cholesterol.

## Data Availability

All data were obtained from the database of the National Health Insurance Sharing Service (NHISS) and are available at https://nhiss.nhis.or.kr/ (accessed 15 November 2022). The NHISS allows access to all data (downloaded from the website) for any researcher who agrees to follow the research ethics and pays a processing fee.
